# An Herbal Nasal Drop Enhanced Frontal and Anterior Cingulate Cortex Activity

**DOI:** 10.1093/ecam/nep198

**Published:** 2011-04-14

**Authors:** Agnes S. Chan, Mei-chun Cheung, Sophia L. Sze, Winnie W. Leung, Dejian Shi

**Affiliations:** ^1^Neuropsychology Laboratory, Department of Psychology, The Chinese University of Hong Kong, Shatin, N.T., Hong Kong; ^2^Integrative Neuropsychological Rehabilitation Center, The Chinese University of Hong Kong, Hong Kong; ^3^Henan Songshan Research Institute for Chanwuyi, Hong Kong; ^4^Institute of Textiles and Clothing, The Hong Kong Polytechnic University, Hong Kong

## Abstract

The present study examined the neuro-electrophysiological activity of the brain associated with the application of a herbal remedy developed by a Shaolin monk based upon the *Chan* healing principle of clearing the orifices (i.e., the nasal cavities). A repeated-measures design was used. Fourteen normal adults were administered herbal remedy and saline solution intranasally on separate sessions. Two intervals of eyes-closed resting EEG data were obtained individually before and after each administration. Results showed that only the herbal remedy but not the saline solution induced elevation in cordance, an index correlated with cerebral perfusion, in the anterior brain region. In addition, the activity of the anterior cingulate cortex (ACC), as examined by the LORETA analysis, was also increased after the application of the herbal remedy but not saline solution. The present study provided some preliminary evidence suggesting that the herbal nasal drop enhanced the activity of the frontal lobe and ACC. Implications for the potential clinical application of the herbal remedy to treat patients with frontal lobe disorders were discussed.

## 1. Introduction

The traditional Shaolin Buddhism training required the practicing monks to be fluent in Shaolin Kungfu, *Chanwu* (martial arts that focus on training Qi and Mind), and a unique healing method, *Chanyi* (healing approach based upon the principle of unblocking Qi and clearing orifices). The term *Chanwuyi* (*Chan,* martial arts and medicine) has been created to describe the uniqueness of this training. The last author of this article is a Shaolin monk who has been practicing *Chanwuyi* for over 20 years and is renowned in China for having treated many patients with illnesses that seemed untreatable. Some empirical studies have been conducted to evaluate the effectiveness of this method [1, 2] and found positive effects. The first author has also reviewed many cases and interviewed many of the last author's patients including late-stage cancer patients, patients with stroke and children with mental retardation. These patients demonstrated various degrees of improvement, including improved motor function, reduced obsessive behavior and improved life expectancy. One of the treatment methods used by the last author was a herbal formula developed by himself and his master based upon the *Shaolin* healing principle of clearing the orifices, according to which all openings of the body are orifices and the nose is the most important orifice in healing illnesses. Therefore, the herbal formula, in the form of liquid drops, is administered through the nasal cavities for clearing this major orifice.

Initial clinical observations on the herbal nasal drop on patients with different brain disorders, including patients with brain tumor, mental retardation and schizophrenia, have found positive effects. This is especially encouraging as no western drug intervention is presently available for cognitive impairment resulting from brain disorders. Patients being administered the herbal remedy have demonstrated 20%–80% improvement in their conditions [3]. We have been using this herbal nasal drop on patients with different brain disorders for 1 year, and the results are very encouraging. For instance, one patient who has demonstrated frontal lobe dysfunction for almost 3 years resulting from epilepsy and stroke has received the herbal nasal drop treatment for 4 months. At baseline, this patient demonstrated many signs of frontal lobe dysfunction including (i) expressive difficulty (i.e., at most three words per conversation), (ii) impairment in comprehension (i.e., could only execute one-component command), (iii) irritability and bad temper (i.e., uncooperative on tasks) and (iv) blunt affect (i.e., no emotional response to praise or reprimand). After a 4-month treatment with the herbal nasal drop, he showed improvement in communication (COM) and social-and-cognitive (SOC-COG) abilities, as measured by the subscales of the Functional Independence Measure (FIM) [4]. His score increased from 5/14 to 7/14 (40% increment) in the COM subscale and from 4/21 to 7/21 (75% increment) in the SOC-COG subscale. Specifically, after the intervention, his verbal expression was improved and he could produce up to five words per conversation and could execute four-component commands. Overall, his quality of life, as measured by the Quality of Life Index (QoLI) [5], has improved from 3 to 7.5 out of 10 points.

**Figure 1:**
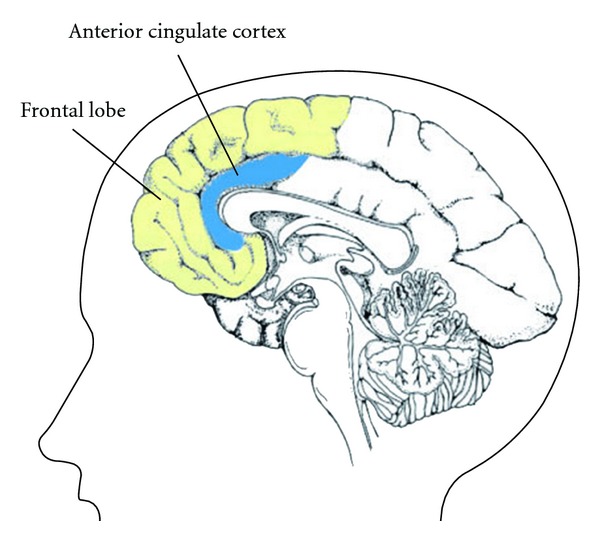
Graphical illustration of the sagittal view of frontal lobe (in yellow) and anterior cingulate cortex (ACC, in blue) in a human brain.

Consistent results were obtained from a pilot study on a group of children with frontal lobe dysfunction who demonstrated impairment on spontaneous speech output, inhibition on repetitive speech and initiation of behavior. Six children were administered herbal nasal drop intranasally for a month, and six age- and intelligence-matched children received a saline solution as control. Treatment-related changes were rated by their parents who were blinded to the group assignment, on an 11-point rating scale (from −5 to +5), where negative rating indicated deterioration, positive rating indicated improvement and zero indicated no change. Initial findings indicated that the treatment group, as compared with the control group, showed significantly greater improvement in spontaneous speech output (mean score: treatment group = 2.24, control group = 0.09, *P* < .01), inhibition on repetitive speech (mean score: treatment group = 1.14, control group = 0, *P* < .05) and initiation of behavior (mean score: treatment group = 2.6, control group = 0.33, *P* < .05). Thus, these encouraging results of our pilot data on both adult and children patients with frontal lobe dysfunction suggested that this herbal formula may have a positive effect on frontal lobe dysfunction. While the clinical trails are on-going, the present study aimed to study the brain activities underlying the intervention effect of this herbal medicine using neuro-eletrophysiological methods. The frontal lobe and anterior cingulate cortex (ACC) (Figure 1) were chosen as two regions of interest as they are significant in mediating frontal lobe function. 

Different methods have been used to study the brain activities and functioning, and quantitative electroencephalography (QEEG) has been one of the widely accepted non-invasive method for this purpose. Two quantitative QEEG measures, cordance [6, 7] and low-resolution electromagnetic tomography (LORETA) [8, 9], have been used to examine brain activities [7, 10–14]. Cordance was developed by Leuchter and colleagues [6] as a non-invasive method that might be used as an index associated with brain perfusion. Brain perfusion reflects the amount of blood flow to the brain tissues, with higher perfusion suggesting higher metabolism in that region. The cordance value was found to be moderately correlated with cerebral perfusion as measured by the positron emission tomography (PET) [6], and this method has been used to study brain activities in patients with depression [15], brain damage [7] and autism [10]. This method was used in the present study to evaluate the effect of the herbal formula on the activity of the frontal region.

The ACC, lying on the medial surfaces of the frontal lobes (Figure 1), is another region of interest in the present study because it is significant in mediating frontal lobe functions. The ACC has been found to have neuronal projections to virtually all areas of the frontal cortex [16] and is functionally connected with the frontal lobe [17]. The functions of ACC were inter-related to that of frontal lobe functions, which has been well documented to mediate cognitive processes involving inhibitory control and monitoring of emotions and behaviors under conflicting situations [18, 19]; the ability to detect erroneous stimuli and response [18]; and efficient allocation of attention to accomplish an effortful cognitive task [19, 20]. Previous findings implicated the ACC as one of the neuronal generators for theta activity in the human brain [14, 21, 22], and LORETA analysis has been one of the common methods used for studying source activity of ACC. Increased theta source activity at ACC localized by the LORETA method has been found to be correlated with increased metabolism at ACC as measured by the PET [23]. LORETA was thus used in this study to examine the effect of the herbal nasal drop on ACC activity of normal adults.

## 2. Method

### 2.1. Study Design and Participants

A repeated-measures design was used with each participant being administered both the herbal formula (experimental condition) and saline (control condition) nasal drops. The order of administration of the two nasal drops was counter-balanced across participants to reduce order effect. The study was performed in accordance with the Helsinki Declaration. Voluntary participation with informed consent was solicited from 14 right-handed normal adults in Hong Kong. Participants (male = 6, female = 8) were aged between 25 and 51 years (mean age = 38.29 years) and had attained university graduate level of education. All participants reported no prior history of head injury, neurologic or psychiatric disorders.

### 2.2. Procedure

EEG was recorded individually in a sound and light-attenuated room during the nasal administration of the herbal formula (experimental condition) or saline (control condition) nasal drops. The two conditions were applied with at least 1 h inter-session interval. In each recording session, the participant lay on his or her back on a bed while the experimenter explained the procedure before he or she was hooked up for EEG recording. A 5-min baseline eyes-closed resting EEG recording was then taken, followed by the application of 10 ml of either the experimental or control agent to the nasal cavities of the participant by the experimenter that lasted for about 5 min. All participants were blinded to the experimental conditions. The two agents were applied with an Eppendorf Multipette plus pipette, in 20 droplets of 0.5 ml each. Then, an eyes-closed resting EEG recording was taken after the administration.

### 2.3. Materials

#### 2.3.1. Herbal Nasal Drop

The herbal nasal drop was provided by the Institute of Chan Herbal Medicine under the Chanwuyi Foundation Limited (a registered charity organization in Hong Kong) and was manufactured under strict Good Manufacturing Practice (GMP) standards. The product has been tested on the level of heavy metal and toxic elements, microbial examination and pesticides residue. The testing results met the product safety guidelines set forth by the Department of Health of the Government of Hong Kong. Some major ingredients include Herba Artemisiae Annuae, Rhizoma Coptidis and Borneol, in a ratio of 1 : 1 : 0.5 and were dissolved in distilled water. All herbal ingredients in the present formula are within the allowed daily dosages as prescribed in the *Chinese Herbal Medicine* published by the Educational Board of the National Drug Administration.

#### 2.3.2. Saline Nasal Drop

A sodium chloride (0.9%) solution commonly used for washing the sinus was used in this study as the control condition.

### 2.4. Data Analysis

Resting EEG data were collected in the eyes-closed condition using an electrode cap with 19 electrode sites (International 10–20 System) referenced to linked ears. The EEG signal was digitized at 256 Hz with a low pass filter of 30 Hz and impedances below 10 kΩ. Body movements were recorded for off-line analyses. EEG data were visually examined for eye movements and muscle artifacts. Only data that had at least 1 min of artifact-free data were selected [24] and spectrally processed using the fast Fourier transformation (FFT) to compute absolute and relative power data for subsequent calculation of the cordance indices. The source of the scalp EEG power measured was localized using the LORETA method. The data were selected and processed by a research assistant who was blinded to the experimental conditions.

Cordance values at theta (4–8 Hz) frequency band was computed for 19 electrode sites (Fp1, Fp2, F3, F4, F7, F8, Fz, T3, T4, T5, T6, C3, C4, Cz, P3, P4, Pz, O1 and O2) according to the methods reported by Leuchter and colleagues [7, 15]. The 19 cordance values were averaged to form three regional mean values, namely the anterior (Fp1, Fp2, F3, F4, F7, F8, Fz and Cz), centrotemporal (T3, T4, T5, T6, C3 and C4) and posterior regions (P3, P4, Pz, O1 and O2). The anterior cordance value was the region of interest in the present study, which may possibly reflect the cerebral perfusion of the frontal lobe. For the LORETA source localization method [8, 9], the sources of the theta band computed from scalp electrical potentials were expressed as 3D cortical current density according to the Talairach brain atlas and were analyzed with a freeware (http://www.uzh.ch/keyinst/loreta.htm). The cordance data were compared using analysis of variance (ANOVA) with repeated measures to examine whether the herbal nasal drop has an effect on brain activities over and above the control condition. LORETA data were also compared between baseline resting and post-nasal drops application using the paired *t*-tests on 2394 voxels with subject-wise normalization process. The aim was to examine whether application of the herbal nasal drop was associated with different sources of scalp EEG as compared with application of the saline solution.

## 3. Results

### 3.1. Analyses on the Activity of the Frontal Cortex

**Figure 2:**
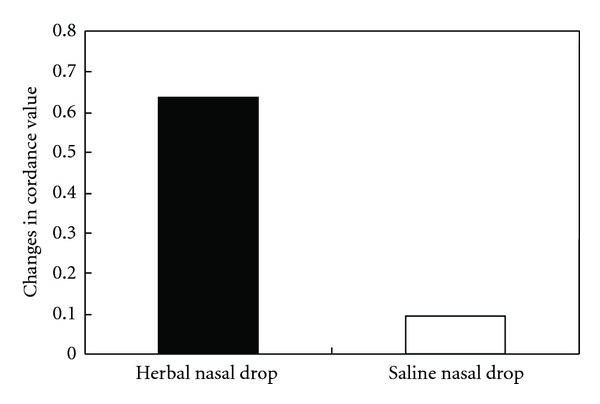
The change in mean anterior cordance values before and after the administration of the herbal nasal drop and saline nasal drop.

A condition (herbal nasal drop versus saline nasal drop) by time (pre- versus post-nasal drop application) repeated-measures ANOVA was performed to compare the theta cordance measured at the anterior brain region. The multivariate results showed a significant interaction effect, *F*(1,13) = 7.01, *P* < .05. Subsequent *post hoc* paired *t*-tests indicated that the anterior cordance value at post-application of the herbal nasal drop was significantly higher than that at the baseline condition (*t*(13) = −3.80, *P* < .01) with a large effect size of 1.01, whereas the difference in anterior cordance values before and after the administration of saline solution was nonsignificant (*t*(13) = −0.76, *P* > .05). Participants' anterior cordance values at the baseline condition before the application of both nasal drops were comparable (*t*(13) = 2.08, *P* > .05). The change in anterior cordance values after the application of the herbal nasal drop (mean increment = 0.64) was significantly greater than that of the saline solution (mean increment = 0.09) (*t*(13) = 2.65, *P* < .05) (Figure 2). Contrasting with the cordance value at the anterior region, both centrotemporal and posterior cordance values did not show similar enhancement in either of the experimental condition (*F* = 0.01 and 3.68, *P* = .93 and .08, resp.). The relatively robust and specific increase in anterior cordance may be suggestive of the effect of the herbal nasal drop in enhancing cerebral blood flow to the frontal lobe. 

To examine the validity of cordance (i.e., whether the effect of the herbal nasal drop is specific to cordance measure or if any EEG measure may yield the same results), other EEG measures were examined. Leuchter and colleagues [7] reported that only EEG cordance, but not absolute and relative power, yielded the strongest relationship with cerebral perfusion. Thus, condition (herbal nasal drop versus saline nasal drop) by time (pre- versus post-nasal drop application) repeated-measures ANOVA analyses were conducted separately for absolute and relative theta power. The mean absolute and relative power was calculated separately by averaging across the electrodes within the anterior scalp regions as that of mean cordance computation. No significant interaction effect was found in either absolute or relative theta power in the anterior brain region (*F* (1,13) = 0.07 and 3.86, *P* > .05), suggesting that the administration of the herbal nasal drop had a specific effect on increasing the cordance level, which is an indicator of enhanced brain activity.

### 3.2. Analyses on the Activity of the Anterior Cingulate Cortex

**Figure 3:**
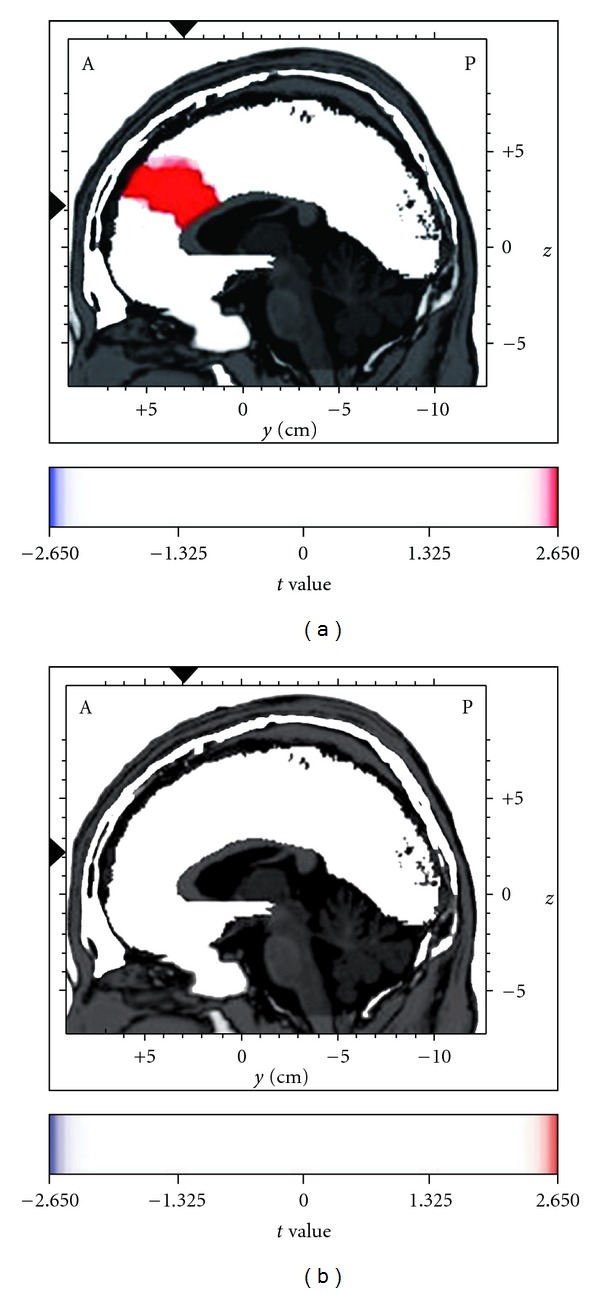
Enhanced theta current density in the anterior cingulate cortex (ACC) after the administration of the herbal nasal drop (a), but not the saline nasal drop (b), as analyzed by the voxel-by-voxel paired *t* statistics with low-resolution electromagnetic tomography (LORETA), *t*(13) = 2.65, *P* < .025. The figure shows the sagittal images at the level of maximal differences between the baseline and the post-intranasal administration time points. The *x*, *y* and *z* Talairach coordinates are −3, 31 and 22, respectively. Red color indicates the source location of significantly increased electrical activity in the brain at post-intranasal administration.

Further to the previous analysis of theta cordance as a measure of the general level of activity of the frontal cortex, we also focused specifically on the activity of the ACC. The LORETA voxel-by-voxel paired *t*-test with subject-wise normalization on the log-transformed data was performed separately for the herbal and saline nasal drop conditions. With repeated tests performed, the alpha was set at 0.025 with Bonferroni adjustment, and the corresponding significant *t-*threshold was at 2.65 (*df* = 13).  Figure 3 shows the graphical representation of the LORETA *t*-statistics separately for each nasal drop condition. It was found that administration of the herbal nasal drop was associated with a significant increase in theta source activity maximally at the ACC (BA 24 and 32) (*t* = 4.02, *P* < .025), while no such increase was observed for the saline condition (*P* = .07). In addition to ACC activation, an increase in theta source activity associated with herbal nasal drop was also found in some frontal structures, including the middle and superior frontal gyri (BA 8 and 10) (*P* < .025). 

Given that the herbal nasal drop and saline solution were applied at separate sessions, the significant theta source activity increment at the ACC associated with the herbal nasal drop may possibly be due to the activity difference at baseline from that of the saline solution. Thus, the same voxel-by-voxel paired *t*-test was performed to compare the baseline theta source activity before the application of the two types of nasal drops. Results indicated that there was no significant difference between the baseline of herbal nasal drop and that of saline solution at the ACC (*t* = 1.79 and 1.87 for BA 24 and 32, resp., *P* > .025). It has, hence, provided further support for the specific effect of the herbal nasal drop on the ACC and implicated that the theta source activity at ACC was relatively stable across time within an individual during baseline resting state.

## 4. Discussion

The primary purpose of the present study was to examine the electrophysiological activities of the brain associated with the administration of a specific herbal nasal drop. Brain activities were recorded before and after nasal application of either the herbal remedy or saline solution. Cordance (an index correlated with brain perfusion) and LORETA (localization of theta source activity of the brain) were used to measure the neural activities of two regions of interest, namely the frontal lobe and the ACC. The results showed that immediately after nasal application of the herbal remedy, the frontal region and the ACC of the brain became more active as evidenced by increased cordance in the frontal region and theta source activity at the ACC. On the other hand, brain activity did not show significant difference before and after the administration of the saline solution. Thus, the results suggested that the specific herbal remedy examined in the present study seemed to be associated with the increasing neural activity.

The present study has provided some insights into the possible mechanism underlying the clinical observation that patients with frontal lobe dysfunction showed improvement after taking the herbal remedy. Specifically, some patients with frontal signs including irritability, expressive difficulty, comprehension impairment and behavioral problems demonstrated various degrees of improvement after 1–4 months of intervention with the herbal remedy. Given that frontal signs are results of frontal lobe and ACC dysfunction, any intervention that increases the activities of these two regions may be able to reduce behavioral impairments due to frontal lobe dysfunction. The present results demonstrated that the nasal application of the herbal remedy increased the activity of the frontal lobe and anterior cingulate cortex, which seems to be explainable for the clinically observed improvements in behavioral problems of patients with frontal lobe dysfunction. Thus, the present findings may have provided initial empirical support for the possibility of using this herbal remedy as potential intervention for enhancing cognitive functions. In fact, an increasing number of studies have found support for the clinical application of herbal medicine as possible complementary medicine for mental disorders [25–35], and the present study has provided additional support.

While administering herbal medicine through the nose has been advocated in the traditional *Shaolin* medical approach, the use of nasal administration of drugs has received increasing attention in the Western medical literature during the last decade. Accumulating evidence shows that administration of drugs through the nasal cavity is especially effective in delivering drugs to the brain [36–38]. An animal study showed that administration of a variant form of dopamine in the right nasal cavity of mice was associated with detection of concentration 27 times higher in the right than the left olfactory bulb [39]. Evidence from human studies also suggested that nasal administration of drugs facilitated the delivery of drugs to the brain. For example, radioactivity in the brain was observed 5 min after the nasal administration of a radioisotope in one study [40], and accumulation of insulin was found in the cerebral spinal fluid after nasal administration of insulin in another study [41]. One study reported that event-related potentials in the brain were changed by nasal administration of drugs [42].

**Figure 4:**
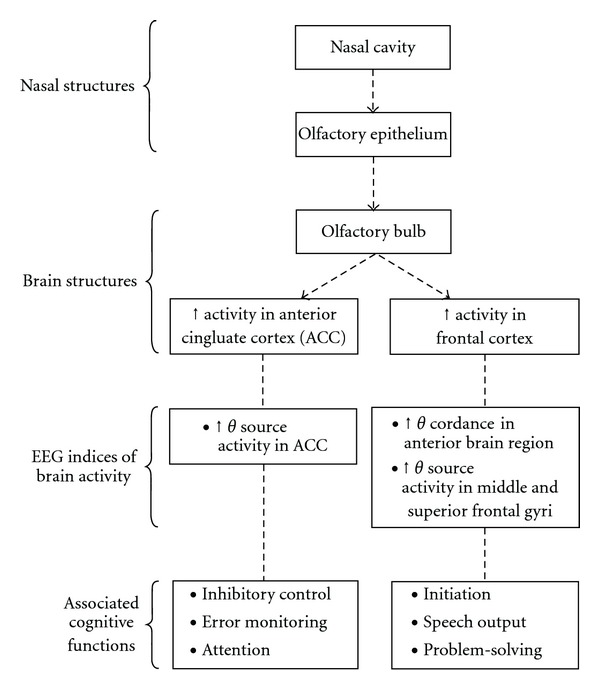
Postulated nose-brain pathway of the effect of the herbal nasal drop on enhancing brain activity and the associated cognitive functions mediated by the frontal and the anterior cingulate brain structures.

The effect of the herbal nasal drop in possibly enhancing cerebral perfusion in the frontal region as reflected by increased theta cordance and increasing source activity at the ACC in normal participants suggests the potential clinical application and therapeutic effect of the herbal nasal drop for treating patients with brain disorders.  Figure 4 shows a schematic representation of the possible pathway of how the herbal nasal drop passes through the nose to influence the brain activity as reflected by the EEG indices, as well as the associated cognitive functions mediated by the frontal lobe and the anterior cingulate brain structures. Intranasal drug administration has the advantages of being noninvasive, rapid in its delivery of drug to the central nervous system, having enhanced drug absorption properties and easy accessibility to blood capillaries. To explore the therapeutic effect of the herbal nasal drop on treating brain disorders, it is worthwhile to examine its effect on patients with well-documented brain dysfunction associated with abnormality in the ACC or frontal lobe. These may include Alzheimer's disease which is related to reduced metabolism and brain atrophy at the cingulate and frontal cortex [43, 44], autistic spectrum disorders and attention deficit/hyperactivity disorder which are related to hypoactivity in the ACC and frontal region [12] and depression which is associated with suppressed activation and perfusion in the left prefrontal cortex [45]. 

The present study has provided initial evidence for the effect of the herbal nasal drop on the physiological state of the brain and on the possible underlying therapeutic nature of this particular herbal formula. However, it has several limitations. First, since the electrophysiological activities were measured immediately after application of the nasal drop, the duration of the effect of the herbal remedy remains to be evaluated in further studies. Second, it remains unclear whether oral administration of the herbal formula, or application of other herbal formulas, will produce a similar effect or not. These questions warrant further investigation. Third, it is by deduction from knowledge of the localization of attentional and inhibitory control abilities to the frontal lobe and ACC region [46, 47] that we postulate that the herbal nasal drop may act on the cognitive functions involving attentional and inhibitory control. Yet, this postulation needs further verification in future studies in which event-related EEG or fMRI assessment will be administered during different cognitive tasks. Fourth, the recording montage adopted in the present study has not included the FPz electrode, which has recently been reported to be specifically sensitive in detecting ACC activity with LORETA [48]. Inclusion of this electrode may thus be considered in the future to explore possible added knowledge about the effect of the herbal nasal drop in ACC. Finally, although the present study and some clinical studies have provided evidence to suggest that the herbal nasal drop may have the potential to become an alternative treatment for brain disorders, randomized controlled trials have to be conducted to establish the effect of the herbal nasal drop on the cognitive functions of patients with brain disorders.

## Funding

This work was supported by the Chanwuyi Foundation Ltd.

## References

[1] Chan AS, Cheung MC, Tsui WJ, Sze SL, Shi D *Dejian* mind-body intervention on depressive mood of community-dwelling adults: a randomized controlled trial.

[2] Chan AS, Sze SL, Shi D (2008). Traditional Chinese mind-body exercises improve self control ability of an adolescent with Asperger's disorder. *Journal of Psychology in Chinese Societies*.

[3] Shi D, Chan AS (2008). *Dejian Mind-Body Intervention: Clinical Application of Shaolin ChanWuYi*.

[4] Keith RA, Granger CV, Hamilton BB, Sherwin FS (1987). The functional independence measure: a new tool for rehabilitation. *Advances in Clinical Rehabilitation*.

[5] Ferrans CE, Powers MJ (1985). Quality of life index: development and psychometric properties. *Advances in Nursing Science*.

[6] Leuchter AF, Cook IA, Lufkin RB (1994). Cordance: a new method for assessment of cerebral perfusion and metabolism using quantitative electroencephalography. *NeuroImage*.

[7] Leuchter AF, Uijtdehaage SHJ, Cook IA, O’Hara R, Mandelkern M (1999). Relationship between brain electrical activity and cortical perfusion in normal subjects. *Psychiatry Research: Neuroimaging Section*.

[8] Pascual-Marqui RD, Michel CM, Lehmann D (1994). Low resolution electromagnetic tomography: a new method for localizing electrical activity in the brain. *International Journal of Psychophysiology*.

[9] Pascual-Marqui RD, Lehmann D, Koenig T (1999). Low resolution brain electromagnetic tomography (LORETA) functional imaging in acute, neuroleptic-naive, first-episode, productive schizophrenia. *Psychiatry Research - Neuroimaging*.

[10] Chan AS, Cheung M-C, Han YMY (2009). Executive function deficits and neural discordance in children with autism spectrum disorders. *Clinical Neurophysiology*.

[11] Chan AS, Han YMY, Cheung M-C (2008). Electroencephalographic (EEG) measurements of mindfulness-based triarchic body-pathway relaxation technique: a pilot study. *Applied Psychophysiology Biofeedback*.

[12] Fallgatter AJ, Ehlis A-C, Seifert J (2004). Altered response control and anterior cingulate function in attention-deficit/hyperactivity disorder boys. *Clinical Neurophysiology*.

[13] Leuchter AF, Cook IA, Witte EA, Morgan M, Abrams M (2002). Changes in brain function of depressed subjects during treatment with placebo. *American Journal of Psychiatry*.

[14] Pizzagalli D, Pascual-Marqui RD, Nitschke JB (2001). Anterior cingulate activity as a predictor of degree of treatment response in major depression: evidence from brain electrical tomography analysis. *American Journal of Psychiatry*.

[15] Cook IA, Leuchter AF, Morgan M (2002). Early changes in prefrontal activity characterize clinical responders to antidepressants. *Neuropsychopharmacology*.

[16] Barbas H (1995). Anatomical basis of cognitive-emotional interactions in the primate prefrontal cortex. *Neuroscience & Biobehavioral Reviews*.

[17] Koski L, Paus T (2000). Functional connectivity of the anterior cingulate cortex within the human frontal lobe: a brain-mapping meta-analysis. *Experimental Brain Research*.

[18] Mulert C, Gallinat J, Dorn H, Herrmann WM, Winterer G (2003). The relationship between reaction time, error rate and anterior cingulate cortex activity. *International Journal of Psychophysiology*.

[19] Sauseng P, Hoppe J, Klimesch W, Gerloff C, Hummel FC (2007). Dissociation of sustained attention from central executive functions: local activity and interregional connectivity in the theta range. *European Journal of Neuroscience*.

[20] Mulert C, Menzinger E, Leicht G, Pogarell O, Hegerl U (2005). Evidence for a close relationship between conscious effort and anterior cingulate cortex activity. *International Journal of Psychophysiology*.

[21] Asada H, Fukuda Y, Tsunoda S, Yamaguchi M, Tonoike M (1999). Frontal midline theta rhythms reflect alternative activation of prefrontal cortex and anterior cingulate cortex in humans. *Neuroscience Letters*.

[22] Ishii R, Shinosaki K, Ukai S (1999). Medial prefrontal cortex generates frontal midline theta rhythm. *NeuroReport*.

[23] Pizzagalli DA, Oakes TR, Davidson RJ (2003). Coupling of theta activity and glucose metabolism in the human rostral anterior cingulate cortex: an EEG/PET study of normal and depressed subjects. *Psychophysiology*.

[24] John ER, Prichep LS, Fridman J, Easton P (1988). Neurometrics: computer-assisted differential diagnosis of brain dysfunctions. *Science*.

[25] Chiappelli F, Navarro AM, Moradi DR, Manfrini E, Prolo P (2006). Evidence-based research in complementary and alternative medicine III: treatment of patients with Alzheimer’s disease. *Evidence-Based Complementary and Alternative Medicine*.

[26] dos Santos-Neto LL, de Vilhena Toledo MA, Medeiros-Souza P, de Souza GA (2006). The use of herbal medicine in Alzheimer’s disease—a systematic review. *Evidence-based Complementary and Alternative Medicine*.

[27] Fu LM, Li JT A systematic review of single Chinese herbs for Alzheimer's disease treatment.

[28] Iwasaki K, Kobayashi S, Chimura Y (2004). A randomized, double-blind, placebo-controlled clinical trial of the Chinese herbal medicine "ba wei di huang wan" in the treatment of dementia. *Journal of the American Geriatrics Society*.

[29] Kitabayashi Y, Shibata K, Nakamae T, Narumoto J, Fukui K (2007). Effect of traditional Japanese herbal medicine toki-shakuyaku-san for mild cognitive impairment: SPECT study. *Psychiatry and Clinical Neurosciences*.

[30] Kum WF, Durairajan SSK, Bian ZX, Man SC, Lam YC, Xie LX Treatment of idiopathic Parkinson's disease with traditional Chinese herbal medicine: a randomized placebo-controlled pilot clinical study.

[31] Maruyama M, Tomita N, Iwasaki K (2006). Benefits of combining donepezil plus traditional Japanese herbal medicine on cognition and brain perfusion in Alzheimer’s disease: a 12-week observer-blind, donepezil monotherapy controlled trial. *Journal of the American Geriatrics Society*.

[32] Radad K, Gille G, Liu L, Rausch W-D (2006). Use of ginseng in medicine with emphasis on neurodegenerative disorders. *Journal of Pharmacological Sciences*.

[33] Seely D, Singh R (2007). Adaptogenic potential of a polyherbal natural health product: report on a longitudinal clinical trial. *Evidence-Based Complementary and Alternative Medicine*.

[34] Suzuki T, Futami S, Igari Y (2005). A Chinese herbal medicine, choto-san, improves cognitive function and activities of daily living of patients with dementia: a double-blind, randomized, placebo-controlled study. *Journal of the American Geriatrics Society*.

[35] Wong AHC, Smith M, Boon HS (1998). Herbal remedies in psychiatric practice. *Archives of General Psychiatry*.

[36] Illum L (2003). Nasal drug delivery—possibilities, problems and solutions. *Journal of Controlled Release*.

[37] Illum L (2004). Is nose-to-brain transport of drugs in man a reality?. *Journal of Pharmacy and Pharmacology*.

[38] Ito N, Nagai T, Oikawa T, Yamada H, Hanawa T Antidepressant-like effect of—perillaldehyde in stress-induced depression-like model mice through regulation of the olfactory nervous system.

[39] Dahlin M, Bergman U, Jansson B, Bjork E, Brittebo E (2000). Transfer of dopamine in the olfactory pathway following nasal administration in mice. *Pharmacological Research*.

[40] Okuyama S (1997). The first attempt at radioisotopic evaluation of the integrity of the nose-brain barrier. *Life Sciences*.

[41] Fehm HL, Perras B, Smolnik R, Kern W, Born J (2000). Manipulating neuropeptidergic pathways in humans: a novel approach to neuropharmacology. *European Journal of Pharmacology*.

[42] Pietrowsky R, Thieman A, Kern W, Fehm HL, Born J (1996). A nose-brain pathway for psychotropic peptides: evidence from a brain evoked potential study with choecystokinin. *Psychoneuroendocrinol*.

[43] DeKosky ST, Scheff SW (1990). Synapse loss in frontal cortex biopsies in Alzheimer’s disease: correlation with cognitive severity. *Annals of Neurology*.

[44] Migneco O, Benoit M, Koulibaly PM, Dygai I, Bertogliati C, Desvignes P (2001). Perfusion brain SPECT and statistical parametric mapping analysis indicate that apathy is a cingulate syndrome: a study in Alzheimer's disease and nondemented patients. *Neuroimage*.

[45] Cook IA, Leuchter AF, Uijtdehaage SHJ (1998). Altered cerebral energy utilization in late life depression. *Journal of Affective Disorders*.

[46] Devinsky O, Morrell MJ, Vogt BA (1995). Contributions of anterior cingulate cortex to behaviour. *Brain*.

[47] Smith EE, Jonides J (1999). Storage and executive processes in the frontal lobes. *Science*.

[48] Korb AS, Cook IA, Hunter AM, Leuchter AF (2008). Brain electrical source differences between depressed subjects and healthy controls. *Brain Topography*.

